# Passive leg raise (PLR) during cardiopulmonary (CPR) – a method article on a randomised study of survival in out-of-hospital cardiac arrest (OHCA)

**DOI:** 10.1186/1471-227X-14-15

**Published:** 2014-07-04

**Authors:** María F Jiménez-Herrera, Youcef Azeli, Eva Valero-Mora, Isaac Lucas-Guarque, Alfonso López-Gomariz, Elena Castro-Naval, Christer Axelsson

**Affiliations:** 1 Department of Nursing, University Rovira i Virgili, Tarragona, Spain; 2 Sistema Emergències Mèdiques de Catalunya, Barcelona, Spain; 3 Borås University, Boras, Sweden; 4 SU Ambulansen Göteborg, Göteborg, Sweden; 5 Departament d’Infermeria, Universitat Rovira i Virgili, Avgda Catalunya, 35, 43002 Tarragona, Spain

**Keywords:** Passive leg raise, Cardiopulmonary resuscitation, Out-of-hospital cardiac arrest, Emergency medical service (EMS)

## Abstract

**Background:**

It is estimated that about 275,000 inhabitants experience an out-of-hospital cardiac arrest (OHCA) every year in Europe. Survival in out-of-hospital cardiac arrest is relatively low, generally between five per cent and 10%. Being able to explore new methods to improve the relatively low survival rate is vital for people with these conditions. Passive leg raise (PLR) during cardiopulmonary resuscitation (CPR) has been found to improve cardiac preload and blood flow during chest compressions. **The aim of our study** is to evaluate whether early PLR during CPR also has an impact on one-month survival in sudden and unexpected out-of-hospital cardiac arrest (OHCA).

**Method/Design:**

A prospective, randomized, controlled trial in which all patients (≥18 years) receiving out-of hospital CPR are randomized by envelope to be treated with either PLR or in the flat position. The ambulance crew use a special folding stool which allows the legs to be elevated about 20 degrees. Primary end-point: survival to one month. Secondary end-point: survival to hospital admission to one month and to one year with acceptable cerebral performance classification (CPC) 1–2.

**Discussion:**

PLR is a simple and fast maneuver. We believe that the greatest benefit with PLR is when performed early in the process, during the first minutes of CPR and before the first defibrillation. To reach power this study need 3000 patients, we hope that this method article will encourage other sites to contact us and take part in our study.

**Trial registration:**

ClinicalTrials.gov NCT01952197.

## Background

The majority of sudden death cases have a cardiac origin and occur unexpectedly, often outside hospital. Attwood et al. [1] estimated the incidence of and survival from EMS-treated OHCA in Europe and found, for “all-rhythm” CA, an incidence of 37.72 per 100,000 person-years. Survival was 10.7% in “all-rhythm” CA. If these results were applied to the European population, approximately 275,000 persons would experience an all-rhythm, EMS-treated OHCA, with 29,000 persons surviving to hospital discharge [1].

To resuscitate a person, without neurological damage, various efforts, which are described as the four links in the chain of survival (early call, early CPR, early defibrillation and early advanced life support), have to be optimal [2]. During the last decade, the quality and continuity of chest compressions have been increasingly highlighted [3]. The reason is that blood flow and coronary perfusion during cardiac arrest are related to the quality and continuity of chest compressions [4]. A coronary perfusion pressure (CPP) above 15 mmHg, at defibrillation, also appears to be necessary for the return of spontaneous circulation (ROSC) [5]. Consequently, different methods and devices to improve blood flow to the heart (coronary perfusion) and brain during CPR, such as different types of mechanical compressor and impedance threshold device [6-9], have been studied.

The initial CPR guidelines [10-12] stated that the “elevation of the lower extremities may promote venous return and augment artificial circulation during external cardiac compression”. However, in the 1992 guidelines [13], this statement was removed. The reason for this decision was a lack of clinical evidence. During the last five years, the debate on how PLR may improve the outcomes of the resuscitation maneuvers in CPR has been re-opened.

According to Préau et al. [14], the effect of PLR is equivalent to a rapid intravenous volume expander by shifting blood from the lower extremities towards the intra-thoracic compartment. A 45° leg elevation for four minutes increases right and left ventricular preload and, by definition, the stroke volume, if the heart is preload dependent [15]. This makes PLR predictive of fluid responsiveness among patients with circulatory failure, e.g. sepsis and acute pancreatitis [14-17], and it has been recommended as part of haemodynamic monitoring in recent international recommendations [18]. Other researchers have also shown the benefit of using PLR to increase resistance to blood flow [19], thereby shifting fluid from the lower extremities to the central circulation [20,21].

The present study design is based on a pilot study recently conducted in Gothenburg, Sweden. This pilot study concluded that a 20° leg elevation during CPR improved the levels of end-tidal carbon dioxide (EtCO_2_) during CPR [22]. It has previously been concluded that EtCO_2_ correlates well with blood flow and that PLR induces an increase in descending aortic blood flow of at least 10% or in echocardiographic sub-aortic flow of at least 12% [23-26]. In other studies, EtCO_2_ has been shown to be quantitatively predictive of stroke volumes [27]. EtCO_2_ has also been described as an important value for predicting ROSC and CPR quality [22,28,29]. The resuscitation in the Gothenburg pilot study was performed using both manual and mechanical compressions made by LUCAS TM 2 (Lund University Cardiac Assist System), but the effect of PLR appeared to be greater during manual compressions. It was only possible to speculate on the reason for this, but the EtCO_2_ value started from a higher level in the mechanical group. The possible reason for this could be the “active decompression” creating a larger preload.

Dragoumanos et al. [30] found in their animal study that the coronary perfusion pressure (CPP) also increased when PLR was performed during CPR and auto-transfusion of the aorta by PLR was the explanation. It is unclear whether this mechanism can be transferred to humans. The literature also includes some case reports and letters advocating PLR during CPR [31,32]. However, no studies showing that PLR during CPR will increase survival have been conducted.

### Hypothesis

The early elevation of the lower extremities during out-of-hospital cardiopulmonary resuscitation increases survival to one month by improving cardiac preload and blood flow to the heart and brain during chest compression.

## Design/method

### Population/area

Tarragona is a province in eastern Spain, in the southern part of the autonomous area known as Catalonia. The district of Tarragona has 814,000 inhabitants in an area of 6,303 km^2^. The population density is 128.2 inhabitants/km^2^ and 17.4% of the inhabitants live in the City of Tarragona and the surrounding areas. The rest are scattered throughout the province, which is composed of 183 municipalities distributed in ten areas. Coastal populations multiply their usual population by five during the months of July and August. The average age of the population is 40 years and 50.49% are men. There is a great deal of scatter in terms of the population throughout the area because of the large rural area that exists there.

### Organisation

Depending on the season, the Emergency Medical Service (EMS) system in Tarragona has around 70 units divided into eight advanced life support (ALS) units, including one helicopter, and 61 basic life support (BLS) units. The ALS units are staffed by one nurse, one physician and one technician and the BLS units by two technicians. Normally, the BLS units are the first responders in health emergencies, e.g. cardiac arrest. The Region of Tarragona has eight public hospitals, located around the region, with different levels of specialities and the highest levels are located in the City of Tarragona.

### Method and design

A prospective, randomized, controlled trial in which all patients (≥18 years) receiving out-of hospital CPR are randomized by envelope to be treated with either PLR or in a flat position. The ambulance crew use a special folding stool, which allows the legs to be elevated about 20 degrees.

The PLR manoeuvre needs to be performed immediately (within five minutes) after the arrival of the first ambulance. Leg elevation has to be maintained while the patient receives chest compressions during CPR and has to be stopped when the patient has an ROSC or when a medical decision is made to interrupt these maneuvers. PLR is to be performed at an angle of between 20 and 45 degrees (approximately 35 to 40 cm). An instruction video is produced for training prior to the study; the aim of using a specially designed folding stool is to standardise the intervention as much as possible.

In the start-up phase between June 2013 and April 2014, the study has only been conducted in the City of Tarragona and the surrounding areas. In all, 13 mobile units (12 BLS and one ALS unit) will attend (attended) in the start-up phase. Since April 2014, a further 56 units, the whole province, have been participating in the study. The study will continue for three years.

## Patient selection and randomization

### Inclusion/exclusion

#### Inclusion

All patients of both sexes who suffer an out-of-hospital cardiorespiratory arrest and require CPR and who are attended by the BLS and/or ALS units in the Tarragona area.

#### Exclusion

Patients aged < 18 will be excluded from the study.

Allocation concealment is ensured via opaque, numbered and sealed envelopes. The random allocation lists are generated by a web-based automated randomization system. To guarantee a numeric balance across conditions the randomization will be performed separately in random permuted blocks of hundred. The allocation list will be kept in a remote secure location and an independent person randomly allocates the envelopes.

### Sample size, preliminary results and study period

**Figure 1:**
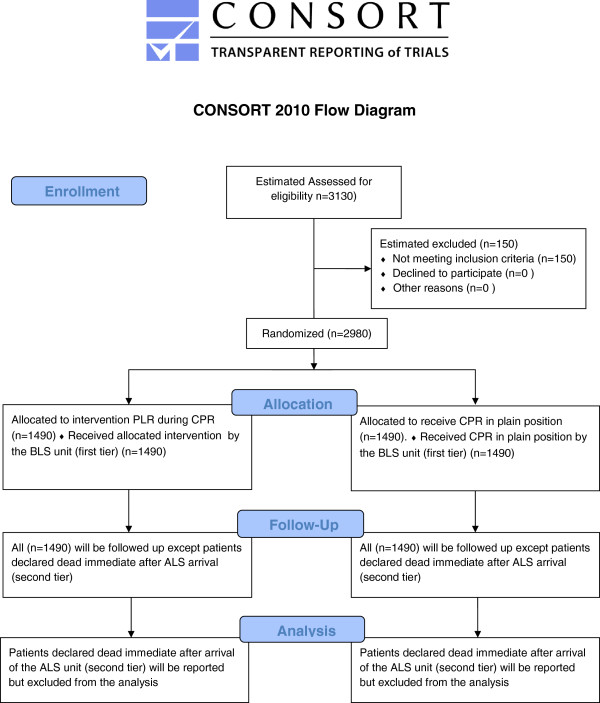
CONSORT 2010 flow diagram.

Survival rates after OHCA in Spain vary greatly, according to different published results. Subsequently, survival to one month and one year in the cohort of OHCA also varies. As a result, the sample size will be estimated from a survival level of seven per cent. However, if we estimate increased survival to one month from seven per cent to 10%, we need 2 × 1,490 patients, a flow diagram of the study is shown in Figure 1. In the intervention group to demonstrate this with 80% power and two-tailed significance of 5%. If the Tarragona district treats 40/100,000 inhabitants, we could plan to include around 300 patients/year in both groups. We plan to continue the study until December 2016 and include two more districts to realise our goal.

### Outcome

Primary end-point: survival to one month.

Secondary end-point: survival to hospital admission to one month and to one year with acceptable cerebral performance classification (CPC) 1–2 [33].

Sub-group analysis: the result will also be analysed in relation to rhythm (shockable/non- shockable rhythm), age (more and less than 65 years), gender and ambulance delay (more and less than 10 minutes).

### Statistical methods

#### Descriptive statistics

The distribution of variables will be given as means, medians and percentages.

### Statistical analysis

Group comparisons (PLR/flat position) will be performed using Fisher’s non-parametric permutation test, the Mann–Whitney U test for continuous/ordered variables and Fisher’s exact test for dichotomous variables.

All tests will be two-tailed and p-values below 0.05 will be considered statistically significant.

### Data registration

The data registration will take place according to “Utstein style” [34] and will be linked to the existing web-based Swedish OHCA Registry. A two-step study protocol, translated into Spanish, is linked inside the Swedish OHCA Registry: part one is completed by the Spanish EMS staff and part two by the study co-ordinator, while part two will comprise follow-up patients admitted alive to hospital. The different parts have different security levels and log-ins. The data management and statistical analysis will be performed in Sweden by an independent statistician.

### Study protocol, variables, part one (EMS)

Ambulance journal number, district, ambulance unit, date.

Randomization: yes (randomization number, PLR Y/N), no (forgot, <18 years, rigor mortis).

Time: of CA, call to dispatch centre, alert ambulance, start ambulance, arrival address, arrival at patient’s side, start CPR, first defibrillation, ROSC time, start transport to hospital, arrival hospital.

Patient data: age, gender, date of birth.

Data on the OHCA incident:

a) Place of occurrence (home, street…)

b) Witnessed: yes (bystander, ambulance staff), no

c) CPR before ambulance arrival: yes (layperson etc.…), no.

d) If CPR before ambulance: defibrillation Y/N (number), ventilation Y/N, chest compression Y/N, telephone CPR Y/N.

e) Status at ambulance arrival: unconscious Y/N, breathing (normal, agonal, no), pulse Y/N.

f) Initial rhythm:

g) Semi-automatic defibrillator: (defibrillation, no defibrillation).

h) If information about rhythm: (VF, VT, PEA, asystole).

i) Reason for CA: (cardiac cause, intoxication, drowning, suicide, accident, pulmonary disease, infant death, asphyxia, other reason).

j) Treatment: (chest compression, mechanical compression, ventilation, intubation, defibrillation (number), epinephrine, atropine, amiodarone, hypothermia.

k) Result: return of spontaneous circulation (ROSC), to hospital Y/N (if yes, which hospital).

l) If arrival hospital: pulse-giving rhythm Y/N, conscious Y/N.

### Study protocol, variables part two (follow-up)

Admitted to hospital ward (yes, no, don’t know).

Received any of following interventions: ICD, PCI/PTCA, CABG (yes, no, planned, don’t know), hypothermia, beta-blocker (yes, no, don’t know).

Discharged alive from hospital: (yes, no, don’t know).

If yes, discharged to: (home, other hospital, other, don’t know).

If yes, discharge date (xx-xx-xx, don’t know).

If yes, CPC score at discharge (1–5, don’t know).

Death within 30 days of CA: (yes, no, don’t know).

If yes, date of death (xx-xx-xx, don’t know).

Follow-up completed ( ).

#### CPC (Cerebral Performance Categories) score

•CPC 1 (no disability).

•CPC 2 (slight disability).

•CPC 3 (moderate disability).

•CPC 4 (comatose/vegetative state).

•CPC 5 (death).

### Data collection

The main variables will be collected by EMS staff from ambulance records. Ambulance delay will be collected from dispatch records. Survival data and CPC score will be collected from hospital records. CPC score will be assessed by health care personal at hospital, blinded to the study protocol. To minimise missing data, the study protocol (database) will be compared with dispatch records. Personal data collected and stored for the purposes of this study will be treated pursuant to the applicable paragraphs of the Organic Law of Personal Data Protection 15/1999, dated 13 December.

### Clinical report form (CRF)

1. Staff member 1: confirm CA and start immediate chest compression.

2. Staff member 2: open defibrillator bag (with envelope and PLR reminder glued to the defibrillator).

3. Staff member 2: open envelope and randomize to PLR or flat position.

4. Staff member 2: if PLR, use the folding stool in the rescue bag.

5. Staff member 2: ventilate the patient once and attach defibrillation pads.

6. Continue resuscitation according to guidelines.

7. If PLR: keep the position as long as the patient is receiving chest compressions.

PLR has to be performed during the first minutes. If PLR is performed later than five minutes after the start of CPR: mark “forgot” on the data sheet.

Web registration: fill in the study protocol for all patients aged ≥18 years receiving out-of-hospital CPR. If not included in the study: just fill in the first part.

### Education/time plan

#### First semester 2013

Introduction of the study, CRF video and basic training of the ALS and BLS teams in the City of Tarragona and surrounding areas. The study was started in Tarragona and surrounding areas on 1/6/2013.

#### First semester 2014

Introduction of the study, CRF video, web journal and basic training of the remaining ALS and BLS teams in the district of Tarragona. The study started in the remaining part of the district on 1/4/2014.

### Ethical approval

The study has the ethical approval of the Ethical Research Committee in Tarragona (CEIC 15/2013) and Reus (13-04-25/4aclaobs1).

## Discussion

The Swedish pilot study [22] was approved by the ethics committees at Stockholm and Gothenburg Universities in March 2003. However, when we applied for new approval in 2011, the study was rejected due to the lack of informed consent. At the moment, there is a debate in Sweden about the opportunity to perform studies of different treatments on unconscious patients. One important issue in the debate is that clinicians find it difficult, time consuming and unethical to ask next of kin, who are upset, for their informed consent in acute situations.

Before we started the present intervention in Spain, we performed our power analysis according to an estimated survival of 7%. In their reports from Spain, Rosell Ortiz [35] and Alvarez Fernandez [36] stated a survival rate of around 10% and an incidence of 25/100,000 EMS-treated OHCAs. Bystander CPR was reported to be performed in about 15% of all cases. Compared with Sweden, the survival rate was similar (10%), but the incidence of EMS-treated OHCA was twice as high (50/100,000) in Sweden [37]. The reason for this can only be speculated on, but shorter ambulance delay in combination with a higher proportion of bystander CPR in Sweden (68%) might be an explanation. Another possible explanation could be the higher competence level in the Spanish ALS units. Compared with the Swedish nurse-staffed ambulances, the Spanish physician-staffed ALS units might have the opportunity to cease resuscitation more frequently [38].

The Swedish pilot study revealed increased EtCO_2_ during PLR, which we interpret as improved cardiac preload and blood flow to the heart and brain during chest compressions. The benefit was greatest when the rescuer performed manual chest compressions [22]. The previous study of PLR was made at an angle of 20 degrees, but, in the present study, we decided to allow the angle to range from 20 to 45 degrees to make the clinical application more flexible. However, the chief researcher (CR) in Tarragona has equipped all the units with a folding stool made of plastic to enable the legs to be elevated 22 centimetres (about 20 degrees).

### Limitations

The power calculation is based on an estimated survival rate of 7%. If the survival rate is lower, we will need a larger sample than calculated. With regard to the outcome of the treatment during OHCA, it may be that the chance of survival does not increase. In no case, however, is there any risk to the participants.

## Competing interests

The authors declare that they have no competing interests.

## Authors’ contributions

The project will be led by the Department of Nursing at the URV in Tarragona and will include researchers from the Department of Statistics and Mathematics and the Department of Basic Medical Sciences, as well as clinical practitioners with extensive experience of cardiopulmonary resuscitation (CPR) research and practice and experts in the field from the University of Borås, Sweden. All authors read and approved the final manuscript.

## Authors’ information

The authors are affiliated to the Rovira i Virgili University, Sistema de Emergencies Mèdiques, Spain, and Borås University and the Gothenburg EMS System, Sweden.

## Pre-publication history

The pre-publication history for this paper can be accessed here:

http://www.biomedcentral.com/1471-227X/14/15/prepub
